# Supplementary material for: Treatment of fatigue with physical activity and behavioural change support in vasculitis: study protocol for an open-label randomised controlled feasibility study
Source: BMJ Open. 2018 Oct 30;8(10):e023769. doi: 10.1136/bmjopen-2018-023769 (PMC6224747; doi:10.1136/bmjopen-2018-023769)
Supplement: Author's manuscript [file bmjopen-2018-023769.draft_revisions.pdf]

BMJ Open is committed to open peer review. As part of this commitment we make the peer review history of every article we publish publicly available.

When an article is published we post the peer reviewers' comments and the authors' responses online. We also post the versions of the paper that were used during peer review. These are the versions that the peer review comments apply to.

The versions of the paper that follow are the versions that were submitted during the peer review process. They are not the versions of record or the final published versions. They should not be cited or distributed as the published version of this manuscript.

BMJ Open is an open access journal and the full, final, typeset and author-corrected version of record of the manuscript is available on our site with no access controls, subscription charges or pay-per-view fees (http://bmjopen.bmj.com).

If you have any questions on BMJ Open's open peer review process please email info.bmjopen@bmj.com

BMJ Open

Treatment of Fatigue with physical activity and behavioural change support in Vasculitis – a feasibility study.

Journal:	*BMJ Open*
Manuscript ID	bmjopen-2018-023769
Article Type:	Protocol
Date Submitted by the Author:	23-Apr-2018
Complete List of Authors:	Harper, Lorraine; University of Birmingham<br>Morgan, Matthew; University of Birmingham<br>Chanouzas, Dimitrios; University of Birmingham<br>Caulfield, Hollie; University of Birmingham<br>Coughlan, Linda; University Hospitals Birmingham NHS Foundation Trust<br>Dean, Caroline; patient participant<br>Fletcher, Kate<br>Cramp, Fiona; The University of the West of England, Allied health professionals<br>Greenfield, Sheila; University of Birmingham, Primary Care Clinical Sciences<br>Hewitt, Catherine; University of Birmingham, Clinical Trials Unit<br>Ives, Natalie; University of Birmingham, BCTU<br>Jowett, Sue; University of Birmingham, Health Economics Unit<br>Daley, Amanda; Loughborough University, School of Sport, Exercise and Health Sciences
Keywords:	vasculitis, fatigue, exercise, anca, self-management

SCHOLARONE™
Manuscripts

For peer review only - http://bmjopen.bmj.com/site/about/guidelines.xhtml

Feasibility study – FAB-V

Treatment of Fatigue with physical activity and behavioural change support in Vasculitis – a feasibility study.

L. Harper[1], M.D. Morgan[1], D. Chanouzas[1], H Caulfield[2], L Coughlan[3], Caroline Dean[4], K Fletcher[2], F Cramp[5], S Greenfield[6], CA Hewitt[7], NJ Ives[7], S Jowett[6], A Daley[8].

1. Institute of Clinical Sciences, College of Medical and Dental Sciences, University of Birmingham, Birmingham B15 2TT, UK

2. Institute of Translational Medicine, College of Medical and Dental Sciences, University of Birmingham, Birmingham B15 2TT, UK

3. NIHR/Wellcome Trust Clinical Research Facility, UHB NHS Foundation Trust, Birmingham B15 2TH, UK

4. Patient Research Partner

5. Faculty of Health and Applied Sciences, University of the West of England Bristol, Bristol BS16 1DD, UK

6. Institute of Applied Health Research, College of Medical and Dental Sciences, University of Birmingham, Birmingham B15 2TT, UK

7. Birmingham Clinical Trials Unit, College of Medical and Dental Sciences, University of Birmingham, Birmingham B15 2TT, UK

8. School of Sport, Exercise and Health Sciences, Loughborough University, Loughborough LE11 3TU, UK

Address for correspondence

Professor Lorraine Harper, Institute of Clinical Sciences, College of Medical and Dental Sciences, University of Birmingham, Birmingham B15 2TT, UK

Email: L.harper@bham.ac.uk

For peer review only

Abstract

Introduction

Fatigue is a major cause of morbidity, limiting quality of life, in patients with ANCA-associated vasculitis (AAV). The aetiology of fatigue is multi-factorial; biological and psychosocial mediators, such as sleep deprivation, pain and anxiety and depression, are important, and may be improved by increasing physical activity. Current self-management advice is based on expert opinion and is poorly adhered to. This study aims to investigate the feasibility of increasing physical activity using a programme of direct contact and telephone support, to provide patient education, encourage behaviour self-monitoring and the development of an individual change plan with defined goals and feedback to treat fatigue compared to standard of care to inform the design of a large randomised controlled trial to test the efficacy and cost-effectiveness of this programme.

Methods and Analysis

Patients with AAV and significant levels of fatigue (multi-dimensional fatigue index score≥14) will be randomised in a 1:1 ratio to the physical activity programme supported by behavioural change techniques or standard of care. The intervention programme will consist of eight visits of supervised activity sessions and 12 telephone support calls over 12 weeks with the aim of increasing physical activity to the level advised by government guidelines. Assessment visits will be performed at baseline, 12, 24 and 52 weeks. The study will assess the feasibility of recruitment, retention, the acceptability, adherence and safety of the intervention and collect data on various assessment tools to inform the design of a large definitive trial. A nested qualitative study will explore patient experience of the trial through focus groups or interviews.

Ethics and Dissemination

All required ethical and regulatory approvals have been obtained. Findings will be disseminated through conference presentations, patient networks and academic publications.

Trial registration number: Prospectively registered ISRCTN 11929227

Strengths and limitations of this study

- Fatigue is a major cause of poor quality of life in patients with ANCA- associated vasculitis, and has no evidence base to direct treatment. This study will provide the evidence required

For peer review only

to design a large randomised controlled trial to address whether physical activity improves fatigue in AAV patients.

- The study is limited by its size; as a feasibility study it has a small sample size and will not provide evidence of efficacy but will provide estimates to calculate sample size for a larger study

For peer review only

Introduction

Fatigue is a common symptom limiting quality of life in patients with a wide range of inflammatory and other chronic diseases including systemic vasculitis(1). Ninety two percent of patients with anti-neutrophil cytoplasm antibody associated vasculitis (AAV) consider fatigue as the most important symptom affecting their well-being(1, 2), and it has been causally linked to reduced social participation, social withdrawal and unemployment(3, 4). Despite the significance of the problem, there are currently no recommended therapies specifically for fatigue in patients with AAV. Current self-management advice provided by the National Institute for Health and Care Excellence (https://cks.nice.org.uk/tirednessfatigue-in-adults#!scenariorecommendation:1) and Arthritis Research UK (ARUK) (https://www.arthritisresearchuk.org/arthritis-information/daily-life/fatigue.aspx) is based on expert opinion and pragmatic advice without any underpinning evidence base. This is therefore, a major area of unmet need and has been identified as a priority research area by patients and ARUK.

The aetiology of fatigue is multi-factorial(5, 6) and related to a number of interacting central (such as reduced motivation and increased perception of effort) and peripheral (impaired muscle or cardiovascular function) biological and psychosocial mediators including sleep deprivation, pain, depression, lack of physical activity and reduced cardiovascular fitness, similar to other chronic diseases (7-9). Fatigue, mainly due to central factors including increased perception of effort, limits physical activity in patients with AAV(10). Only 47% of AAV patients participate in at least one hour of moderate or vigorous physical activity per week (unpublished audit of 100 patients) and patients are reluctant to increase physical activity often due to fatigue, or for fear of worsening their fatigue(11). Both central and peripheral components of fatigue need to be addressed in any successful treatment intervention.

Physical activity interventions improve sleep, quality of life, anxiety and depression in patients with chronic diseases, and have been shown to improve fatigue in the short term(12). However, studies with long term follow up often report no sustained increase in physical activity once support is discontinued(13). The addition of cognitive behavioural support (CBS), as commonly implemented in successful chronic disease self-management programmes(14), to a physical activity intervention may result in improved self-efficacy and a sustained increase in physical activity once the formal programme is completed(15-18).

For peer review only

Our programme, using direct contact and telephone support, will provide patient education, encourage behaviour self-monitoring and the development of an individual change plan with defined goals and feedback to increase physical activity. These interventions have not been investigated to treat fatigue in patients with systemic vasculitis. A pilot study of five AAV patients with fatigue suggested physical activity, supported by behavioural intervention, was an acceptable approach for patients with AAV and fatigue(19).

Aims and Objectives

The aim of this study is to assess the feasibility and acceptability of undertaking a phase III randomised controlled trial (RCT) of physical activity with behaviour change support including device assisted self-monitoring and telephone support with cognitive behavioural strategies compared to standard of care in patients with AAV.

The specific objectives of the study are to:

- Assess different recruitment methods to the trial e.g. where and how patients are identified and recruited
- Assess recruitment and retention rates in the trial
- Assess the acceptability of the intervention and the burden of trial participation on patients
- Assess adherence to the trial intervention
- Assess the safety of the intervention with respect to possible injury and deterioration in fatigue levels as a result of participating in a physical activity trial
- Assess the effect of trial participation on activity levels in patients in the standard care group
- Test procedures for the trial, including delivery of the standardised protocolled intervention and administration of trial questionnaires and other outcome measurement tools, and identify areas that may improve the design and implementation of the intervention
- Collect data on the variability of the outcome measures to inform a power calculation for a definitive trial
- Provide an estimate of the cost of implementing the intervention
- Collect feedback from patients on their experience of participating in the study

For peer review only

Methods

Overview of study design

This is a single centre, open-label randomised controlled feasibility study with a nested focus component. Feasibility will be determined by assessing recruitment, adherence to the intervention and retention rates. Acceptability and patient experience will be assessed through patient reported outcome questionnaires (PROQ), and an optional nested qualitative study consisting of either focus groups or semi-structured telephone interviews if insufficient numbers can be recruited to form focus groups..

Fifty participants will be randomised (via a computer generated programme) in a 1:1 ratio to the intervention or standard care for this population (Figure 1 study flow). The randomisation will be minimised by age (<65 vs. ≥65 years). Participants randomised to the intervention arm will complete eight supervised (one per week) and 12 telephone coaching sessions over 12 weeks. Outcomes will be assessed at baseline, 12, 24 and 52 weeks from the first exercise session for those in the intervention group and from randomisation for those in the standard care group.

Sample size

This is a feasibility study and therefore a formal sample size calculation has not been performed. The study is not designed or powered to detect a statistically significant difference in efficacy between the two treatment arms.

A pragmatic recruitment target of 50 participants has been chosen as we expect this number will be feasible within a single centre with a population of 250 patients and should be sufficient to provide estimates of the feasibility outcomes. A sample size of between 24 and 50 is recommended for pilot studies to estimate sample sizes for a full trial(20).

Patient Involvement

The study was designed in consultation with a range of patients. A patient research partner (CD) is part of the trial management group and helped design the study which was then discussed at patient support group meetings and with trustees of Vasculitis UK, the patient support charity. Feedback from these meetings was incorporated into the study design. Vasculitis UK has helped with patient recruitment and will help with dissemination of the study results.

Participants and recruitment

For peer review only

Patients will be recruited to the study via the dedicated vasculitis service at University Hospitals Birmingham NHS Foundation Trust (UHBNFT) or via response to study adverts placed in the Vasculitis UK newsletter or other hospital clinics. Confirmation of eligibility for the study will be with the patient's own clinician. Patients are eligible if they are 18 years of age or older, have a diagnosis of AAV as classified by the European Medicines Agency Algorithm (EMEA)(21), have been in remission for at least six months on the day of consent (defined by Birmingham Vasculitis Activity Score Version 3 (BVASv3)(22)=0 on day of consent AND prednisolone dose <7.5mg for 6 months) and have significant fatigue levels (multi-dimensional fatigue inventory (MFI)-20 general fatigue score ≥14). Exclusion criteria include inability to provide informed consent, inability or unwillingness to undertake physical activity, co-morbidities considered by their own clinician to contra-indicate an increase in physical activity and an inability to understand and complete questionnaires.

Standard Care Group

Patients randomised to the standard of care (control) group will receive their standard clinical care and will be provided with verbal activity advice as per UK government recommendations. Advice on activity will be given after randomisation and patients will be advised to visit the government physical activity guidelines website(23)

Intervention Group

The intervention is a physical activity and behavioural change support programme, plus standard care. The physical activity intervention will provide support for 12 weeks; weeks 1-8 will consist of weekly (where possible) direct contact in groups of 4-7 patients and additional individual telephone health coaching once weekly (where possible), then in weeks 9-12 telephone health coaching only will be provided. The intervention will be staged-adapted and participants will be encouraged to increase physical activity within their capabilities. Participants will be provided with personal activity self-monitoring devices, which are wrist worn accelerometers (Fitbit Model FB405BKL), providing individual feedback on daily physical activity. The supervised activity sessions will incorporate cognitive-behavioural strategies (e.g. goal setting, finding social support and understanding the costs/benefits of exercise etc.) to promote long-term participation in physical activity. The intervention includes education, monitoring and assessment of progress and teaches skills with the aim of increasing self-efficacy.

Individual SMART (specific, measurable, achievable, realistic and timed) goals(24) will be set by the participants with guidance from the intervention facilitator and will focus on increasing activity

For peer review only

levels. The behavioural goal is for participants to achieve at least 30 minutes of moderate intensity activity, 5 days per week, ideally in bouts of 10 minutes, as per UK government recommendations, although any increase in physical activity or reduction in sedentary time will be viewed positively. The Fitbit activity self-monitor is supported with online web-interfaces that facilitate effective components of physical activity interventions (https://www.fitbit.com/login)(25).

A detailed facilitator intervention manual has been developed to support the supervised sessions and telephone coaching. In brief the physical activity intervention is designed to be pragmatic and accessible, taking into account exercise preferences and giving choices. During the direct contact sessions participants will be asked to complete short bouts (e.g. 5 × 3 minutes, with two-minute rest intervals) of low to moderate intensity aerobic exercise (e.g. stepping ergometer, cycle-ergometer, treadmill walking, rowing ergometer, arm-cranking) at 50%–69% of predicted maximum heart rate (220–age) or 12–14 on the Borg Ratings of Perceived Exertion Scale(26). Intensity will be monitored continuously during exercise training sessions. As the intervention progresses, participants will be encouraged to participate in longer periods of aerobic exercise (e.g. 5 × 4 minutes) or to take shorter rests between bouts.

Where appropriate, participants will also be introduced to exercises for strength and control, which will typically involve two to six different resistance exercises (e.g. wall press-ups, arm-curls, leg abduction, wall squats and/or regular squats, knee extensions, calf raises, sit-to-stand) each session. Body resistance, light weights and *Therabands* will be used to provide resistance and one to three sets of five to 20 repetitions will be performed, depending on level of disability and strength, as well as stage of the programme (exercises will be progressed according to individual capabilities and strength gains). Static stretching exercises for large skeletal muscle groups will be included in the sessions.

Participants will be encouraged to replicate the sessions at home supported by the telephone health coaching.

Using activity data collected from the Fitbit device from the preceding week, the intervention facilitator will assist participants to establish SMART goals(24). Data collected will be shared with the therapist, via either the internet dashboard or paper diary. Review of daily and weekly physical activity will be undertaken to identify bouts of moderate/vigorous physical activity and to negotiate change plans to increase bouts of moderate/vigorous physical activity.

For peer review only

During the telephone discussion, participants will be encouraged to use their activity profiles generated from their Fitbit device, with data collected and stored using the online dashboard or paper diaries, to promote self-determination and self-regulation to achieve personal goals and maintain activity. The therapist will review fatigue, the impact of physical activity on fatigue and the management of fatigue with the participant. The therapist will review activity plans and understanding, provide physical activity education, and help the patient generate activity maintenance plans and set new goals.

Study procedures

The study outcomes, measurement methods and assessments are summarised in table 1. Data is collected at baseline, and then at 12, 24 and 52 weeks from the first exercise session for those in the intervention group and from randomisation for those in the standard care group. Data on recruitment, number of activity sessions and telephone coaching sessions completed and drop outs will also be recorded.

For peer review only

Table 1 **Visit overview**

Visit	*Pre-baseline*	*Baseline*	*Baseline (+ 4 weeks)*	*Weeks 1-8*	*Weeks 9-12*	*Week 12**	*Week 24**	*Week 52**
Identification of eligible patients	*x*							
Eligibility check	*x*	*x*						
Valid informed consent		*x*						
Relevant medical history taken		*x*						
Randomisation (without patient present)			*x*					
*Activity advice telephone call (control arm only)***				*x*				
Exercise Visits (Intervention arm only)				*x*				
Weekly Telephone Coaching contacts (Intervention arm only)				*x*	*x*			
*Follow up clinic visits**[$]						*x*	*x*	
*Final assessment (postal questionnaires)**[$]								*x*
Quality of life questionnaires		*x*				*x*	*x*	*x*
Focus Groups or interviews (post intervention)					*x*	*x*	*x*	

******The timing of the follow up visits for intervention group are taken from intervention start date, not randomisation date; for the control group, the timing is taken from randomisation date.*

****** *Control group have one telephone call in week 1 (post randomisation) to receive standard activity advice only.*

[$] *12 and 24 week follow up assessments can be completed up to -2 weeks before due date and up to 3 weeks after the due date*

[$$] *52 weeks follow up assessment can be completed -2 weeks before due date and up to 8 weeks after the due date.*

For peer review only

At the baseline, 12 and 24 week assessments blood pressure, height (baseline only), weight and waist circumference will be measured, disease type, medical and drug history (baseline only), use of immunosuppressive therapy and assessment of damage associated with AAV, using the vasculitis damage index(27) (baseline only) will be taken. Laboratory investigations, including full blood count and kidney function are measured at baseline. At each assessment visit, disease activity will be assessed using the BVASv3. Clinical outcomes, including muscle or bone injury, disease relapse and cardiovascular adverse clinical events will be collected at 12 and 24 weeks. Participants will complete a suite of validated health related quality of life and fatigue questionnaires, which include Short Form-36 (SF-36)(28), ANCA Vasculitis questionnaire (AAV-PRO), EQ-5D(29), MFI-20(30), Pittsburgh Sleep Index(31), Bristol Rheumatoid Arthritis Fatigue- Multi-dimensional questionnaire (BRAF-MDQ)(32), Hospital Anxiety and Depression score (HADS)(33), Brief COPE questionnaire(34) and the International Physical Activity Questionnaire (IPAQ) long version at baseline and at 12, 24 and 52 weeks (at 52 weeks, the participants will be contacted by post and asked to complete the same questionnaires as completed at previous assessments and to return these by post or at next clinic visit). All participants will be asked to wear an accelerometer (GENEActive GATV01, Activinsights, Kimbolton UK) for 7 days at baseline and at 12 and 24 weeks to provide an objective measure of their activity.

In order to undertake an analysis of all the direct resources and costs required to deliver the intervention, a detailed collection of health economic data will be embedded within the trial.

Study outcomes and data analysis

The primary outcome of this feasibility trial is to determine if a full RCT comparing physical activity with behaviour change support versus standard care in patients with AAV is feasible. This decision will be made using a composite assessment of both quantitative and qualitative data based on a traffic light system using pre-defined stop-go criteria. The three criteria contributing to the traffic light system include (1) recruitment rates, defined as the proportion of eligible patients recruited into the study; (2) successful adherence to the intervention, defined as attendance at a minimum of 4 of the 8 visits for the face to face support, and acceptance of a minimum of 3 of the 4 telephone support calls in weeks 9-12 and (3) study drop-out, defined as complete withdrawal from the study, with no further data collected from the participant. The traffic light system comprises 1) **Green light**: recruitment rate >50%; adherence rate >75%, and drop-out rate <15% excluding those patients who drop-out or are unable to achieve adherence due to disease activity. If **all three** criteria are met, we will proceed to a full trial with the protocol unchanged (unless there is a clear message from the

For peer review only

qualitative work that would improve the protocol). 2) **Amber light:** recruitment rate 30-50%, adherence rate 50-75%, or drop-out rate 15-30% excluding those patients who drop-out or are unable to achieve adherence due to disease activity. If **one or more** of our amber light criteria are met, we will adapt the protocol in light of the result of the feedback from the qualitative work and our experience to improve whichever criteria are not at the "green-light" level before proceeding to full trial if possible. We will assess whether adaption of the protocol will require a further feasibility study or pilot study before progressing. 3) **Red light:** recruitment rate <30%, adherence rate <50%, or drop-out rate >30% excluding those patients who drop-out or are unable to achieve adherence for disease activity. If **one or more** of these criteria are met we would consider the current protocol not feasible and not progress to a full RCT.

The primary analysis to assess the stop-go criteria will be undertaken once all participants have completed the 24 week assessment and corresponding outcome data has been entered onto the study database and validated as being ready for analysis. Feasibility outcomes including the three components of the stop-go criteria will be analysed by pooling the two randomised groups and presenting overall estimates with 95% confidence intervals. Reasons for non-entry into the trial will be assessed, particularly in relation to the patient eligibility criteria and reasons for patient refusal. Data on patients who stop the intervention and/or who do not complete the trial (such as withdrawals and those lost to follow-up) will be collected throughout the trial to allow assessment of patient adherence and retention rates. Reasons for non-adherence and non-completion will be analysed descriptively.

All clinical and patient reported outcomes will primarily take the form of simple descriptive statistics (e.g. proportions and percentages, means and standard deviations) and where appropriate, point estimates of effects sizes (e.g. mean differences and relative risks) and associated 95% confidence intervals. Data return rates at each time point will be assessed along with data completeness of the various outcomes measures. The data will be analysed using the intention to treat principle, where patients are analysed in the treatment group to which they were randomised regardless of adherence to intervention or compliance with the protocol.

Detailed data collection of the resources required to deliver the intervention will allow a cost analysis to be undertaken to calculate the cost of implementing the intervention in a full RCT. The mean cost of the intervention per patient will be estimated both as per usage in the study and also

For peer review only

the full cost assuming full compliance. Sensitivity analysis will estimate costs with changes to costing assumptions, e.g. staff grade, group size.

Nested qualitative study

Focus groups of 4-6 participants will be carried out to discuss their experience of the trial. Two groups will consist of people who participated in the trial and participated in the intervention, one group of people who participated and received standard of care; and the final group will be made up of people who did not wish to participate in the trial. Individuals who did not wish to participate in the trial will be contacted and invited to participate in a focus group to better understand the reasons for non-participation. If we cannot form one or more of the focus groups described, then patients will be invited to participate in 1:1 semi-structured interviews The qualitative phase will be conducted by a researcher not involved with the rest of the study and will collect data on the experiences of the study and the intervention in more detail, and to gather suggestions for improvements to the design of a future RCT.

Focus groups and interviews will be recorded and transcribed verbatim. QSR NVivo 8 will be used for data management and data analysed using a framework approach(35), facilitated by the use of to identify freely emerging themes and categories. Those who did not participate in the trial will be invited to complete the same questionnaires as collected during the trial. These will be analysed as above using simple descriptive statistics.

Trial management and monitoring

The trial will be coordinated by the trial management group (TMG) (principal investigator, co-investigators, trial coordinator, physical activity trainer and statistical advisors) in conjunction with the NIHR/WT CRF. A Trial Steering Committee (TSC) consisting of the principal investigator as well as three independent consultants, a statistician and patient advocate not involved in the study will provide the overall supervision of the trial. The TSC will oversee trial progress, protocol compliance, patient safety and review of updated information. As this is a feasibility study with short follow up, no Data Monitoring Committee will be formed, as agreed with the sponsor. The integrity of data entry will be ensured using a trial-specific Data Input Quality Control standard operating procedure (Trial Master File, University of Birmingham). The trial database will contain date of birth and sex. Data analyses will be undertaken on pseudo-anonymised datasets. All source data and original participant identities will be kept in a locked office in the trial site file only at UHB NHSFT or at BCTU.

For peer review only

Ethics

The protocol was reviewed and obtained a favourable opinion by the Health Research Authority (IRAS project ID 210364) on 29/09/16 and adopted on to the NIHR portfolio. All patients will be provided with verbal and written study information and written informed consent will be obtained before participation. The study will be undertaken at a single site, and local governance approval has been granted by UHB NHSFT. Steps have been taken to ensure patient welfare when designing this study. The study complies with the International Conference for Harmonisation of Good Clinical Practice guidelines and the Research Governance Framework for Health and Social Care.

For peer review only

Sponsor

The study is sponsored by the University of Birmingham. The University of Birmingham holds public liability (negligent harm) and clinical trial (negligent harm) insurance policies, which apply to this trial. The sponsor has been involved in protocol design as well as the development of the case report form.

Funding

Funding for the study has been provided by Arthritis Research UK.

Dissemination policy

It is anticipated that the findings of this study will be published in peer-reviewed journals, presented at national conferences and that the results will be disseminated to all study participants who wish to be informed. It is anticipated that the results of this study will inform the design of a large RCT investigating the efficacy of increasing physical activity to treat fatigue in this population.

References

1. Grayson PC, Amudala NA, McAlear CA, Leduc RL, Shereff D, Richesson R, et al. Illness perceptions and fatigue in systemic vasculitis. Arthritis Care Res (Hoboken). 2013;65(11):1835-43.
2. Herlyn K, Hellmich B, Seo P, Merkel PA. Patient-reported outcome assessment in vasculitis may provide important data and a unique perspective. Arthritis Care Res (Hoboken). 2010;62(11):1639-45.
3. Basu N, McClean A, Harper L, Amft EN, Dhaun N, Luqmani RA, et al. Markers for work disability in anti-neutrophil cytoplasmic antibody-associated vasculitis. Rheumatology (Oxford). 2014;53(5):953-6.

4. Carpenter DM, Meador AE, Elstad EA, Hogan SL, DeVellis RF. The impact of vasculitis on patients' social participation and friendships. Clin Exp Rheumatol. 2012;30(1 Suppl 70):S15-21.
5. Basu N, McClean A, Harper L, Amft EN, Dhaun N, Luqmani RA, et al. Explaining fatigue in ANCA-associated vasculitis. Rheumatology (Oxford). 2013;52(9):1680-5.
6. McClean A, Morgan MD, Basu N, Bosch JA, Nightingale P, Jones D, et al. Physical Fatigue, Fitness, and Muscle Function in Patients With Antineutrophil Cytoplasmic Antibody-Associated Vasculitis. Arthritis Care Res (Hoboken). 2016;68:1332-9.
7. Jones DE, Gray JC, Newton J. Perceived fatigue is comparable between different disease groups. Qjm. 2009;102(9):617-24.
8. Hewlett S, Chalder T, Choy E, Cramp F, Davis B, Dures E, et al. Fatigue in rheumatoid arthritis: time for a conceptual model. Rheumatology (Oxford). 2011;50(6):1004-6.
9. Cleanthous S, Tyagi M, Isenberg DA, Newman SP. What do we know about self-reported fatigue in systemic lupus erythematosus? Lupus. 2012;21(5):465-76.
10. Newall C, Schinke S, Savage CO, Hill S, Harper L. Impairment of lung function, health status and functional capacity in patients with ANCA-associated vasculitis. Rheumatology. 2005;44:623-8.
11. Thorpe CT, DeVellis RF, Blalock SJ, Hogan SL, Lewis MA, DeVellis BM. Patient perceptions about illness self-management in ANCA-associated small vessel vasculitis. Rheumatology (Oxford). 2008;47(6):881-6.
12. Yang PY, Ho KH, Chen HC, Chien MY. Exercise training improves sleep quality in middle-aged and older adults with sleep problems: a systematic review. J Physiother. 2012;58(3):157-63.
13. Cramp F, Hewlett S, Almeida C, Kirwan JR, Choy EH, Chalder T, et al. Non-pharmacological interventions for fatigue in rheumatoid arthritis. Cochrane Database Syst Rev. 2013;8:CD008322.
14. Lorig KR, Holman H. Self-management education: history, definition, outcomes, and mechanisms. Ann Behav Med. 2003;26(1):1-7.
15. Hewlett S, Ambler N, Almeida C, Cliss A, Hammond A, Kitchen K, et al. Self-management of fatigue in rheumatoid arthritis: a randomised controlled trial of group cognitive-behavioural therapy. Ann Rheum Dis. 2011;70(6):1060-7.
16. Midtgaard J, Tveteras A, Rorth M, Stelter R, Adamsen L. The impact of supervised exercise intervention on short-term postprogram leisure time physical activity level in cancer patients undergoing chemotherapy: 1- and 3-month follow-up on the body & cancer project. Palliat Support Care. 2006;4(1):25-35.
17. Adamsen L, Quist M, Midtgaard J, Andersen C, Moller T, Knutsen L, et al. The effect of a multidimensional exercise intervention on physical capacity, well-being and quality of life in cancer patients undergoing chemotherapy. Support Care Cancer. 2006;14(2):116-27.
18. Tierney S, Mamas M, Woods S, Rutter MK, Gibson M, Neyses L, et al. What strategies are effective for exercise adherence in heart failure? A systematic review of controlled studies. Heart Fail Rev. 2012;17(1):107-15.
19. N Basu PP, S Lawton, M Greenwood, C Dent, K Kindness, E John, C Arthur, L West, MV Macfarlane, DM Reid, GT Jones, GJ Macfarlane. Multi-disciplinary Management of Fatigue in ANCA Associated Vasculitis: A Pilot Study. BSR. 2012.
20. Sim J, Lewis M. The size of a pilot study for a clinical trial should be calculated in relation to considerations of precision and efficiency. J Clin Epidemiol. 2012;65(3):301-8.
21. Watts R, Lane S, Hanslik T, Hauser T, Hellmich B, Koldingsnes W, et al. Development and validation of a consensus methodology for the classification of the ANCA-associated vasculitides and polyarteritis nodosa for epidemiological studies. Ann Rheum Dis. 2007;66(2):222-7.

For peer review only - http://bmjopen.bmj.com/site/about/guidelines.xhtml

22. Mukhtyar C, Lee R, Brown D, Carruthers D, Dasgupta B, Dubey S, et al. Modification and validation of the Birmingham Vasculitis Activity Score (version 3). Ann Rheum Dis. 2009;68(12):1827-32.
23. https://www.gov.uk/government/uploads/system/uploads/attachment_data/file/213741/dh_128146.pdf.
24. Doran G. There's a S.M.A.R.T. way to write management's goals and objectives. Manage Rev. 1981;70:35-6.
25. Hardcastle SJ, Taylor AH, Bailey MP, Harley RA, Hagger MS. Effectiveness of a motivational interviewing intervention on weight loss, physical activity and cardiovascular disease risk factors: a randomised controlled trial with a 12-month post-intervention follow-up. Int J Behav Nutr Phys Act. 2013;10:40.
26. Borg G. A category scale with ratio properties for intermodal and interindividual comparisons. In: Geissler H, Petzold P, eds. Psychophysical judgement and the process of perception; 1984:25-34.
27. Exley AR, Bacon PA, Luqmani RA, Kitas GD, Gordon C, Savage CO, et al. Development and initial validation of the Vasculitis Damage Index for the standardized clinical assessment of damage in the systemic vasculitides. Arthritis Rheum. 1997;40(2):371-80.
28. McHorney CA, Ware JE, Jr., Raczek AE. The MOS 36-Item Short-Form Health Survey (SF-36): II. Psychometric and clinical tests of validity in measuring physical and mental health constructs. Med Care. 1993;31(3):247-63.
29. Rabin R, de Charro F. EQ-5D: a measure of health status from the EuroQol Group. Ann Med. 2001;33(5):337-43.
30. Smets EM, Garssen B, Bonke B, De Haes JC. The Multidimensional Fatigue Inventory (MFI) psychometric qualities of an instrument to assess fatigue. J Psychosom Res. 1995;39(3):315-25.
31. Buysse DJ, Reynolds CF, 3rd, Monk TH, Berman SR, Kupfer DJ. The Pittsburgh Sleep Quality Index: a new instrument for psychiatric practice and research. Psychiatry Res. 1989;28(2):193-213.
32. Hewlett S, Dures E, Almeida C. Measures of fatigue: Bristol Rheumatoid Arthritis Fatigue Multi-Dimensional Questionnaire (BRAF MDQ), Bristol Rheumatoid Arthritis Fatigue Numerical Rating Scales (BRAF NRS) for severity, effect, and coping, Chalder Fatigue Questionnaire (CFQ), Checklist Individual Strength (CIS20R and CIS8R), Fatigue Severity Scale (FSS), Functional Assessment Chronic Illness Therapy (Fatigue) (FACIT-F), Multi-Dimensional Assessment of Fatigue (MAF), Multi-Dimensional Fatigue Inventory (MFI), Pediatric Quality Of Life (PedsQL) Multi-Dimensional Fatigue Scale, Profile of Fatigue (ProF), Short Form 36 Vitality Subscale (SF-36 VT), and Visual Analog Scales (VAS). Arthritis Care Res (Hoboken). 2011;63 Suppl 11:S263-86.
33. Zigmond AS, Snaith RP. The hospital anxiety and depression scale. Acta Psychiatr Scand. 1983;67(6):361-70.
34. Carver CS. You want to measure coping but your protocol's too long: consider the brief COPE. Int J Behav Med. 1997;4(1):92-100.
35. Gale NK, Heath G, Cameron E, Rashid S, Redwood S. Using the framework method for the analysis of qualitative data in multi-disciplinary health research. BMC Med Res Methodol. 2013;13:117.

Footnotes

Funding

This work was supported by Arthritis Research UK grant number 21199.

For peer review only

Ethical approval

The protocol was reviewed and obtained a favourable opinion by the Health Research Authority (IRAS project ID 210364) on 29/09/16 and adopted on to the NIHR portfolio

Competing interests

All authors have completed the ICMJE uniform disclosure form. No financial relationships with any organisations that might have an interest in the submitted work in the previous three years; no other relationships or activities that could appear to have influenced the submitted work have been declared by the authors.

Contribution

All authors developed and reviewed the protocol. All authors conceived the data analysis plan and the writing of this article. All authors critically reviewed and edited drafts and approved the final version of the manuscript. They also had full access to all of the data (including statistical reports and tables) in the study and can take responsibility for the integrity of the data and the accuracy of the data analysis. The study guarantor is LH.

Data sharing agreement – no additional data available

Provenance and peer review – not commissioned and externally peer reviewed

Acknowledgements

Trustees and patient members of Vasculitis UK are thanked for their helpful contributions which informed the development of the protocol. The work was carried out at the National Institute for Health Research (NIHR)/Wellcome Trust Birmingham Clinical Research Facility. The views expressed are those of the author(s) and not necessarily those of the NHS, the NIHR of the Department of Health.

Figure 1 Consort Diagram

For peer review only

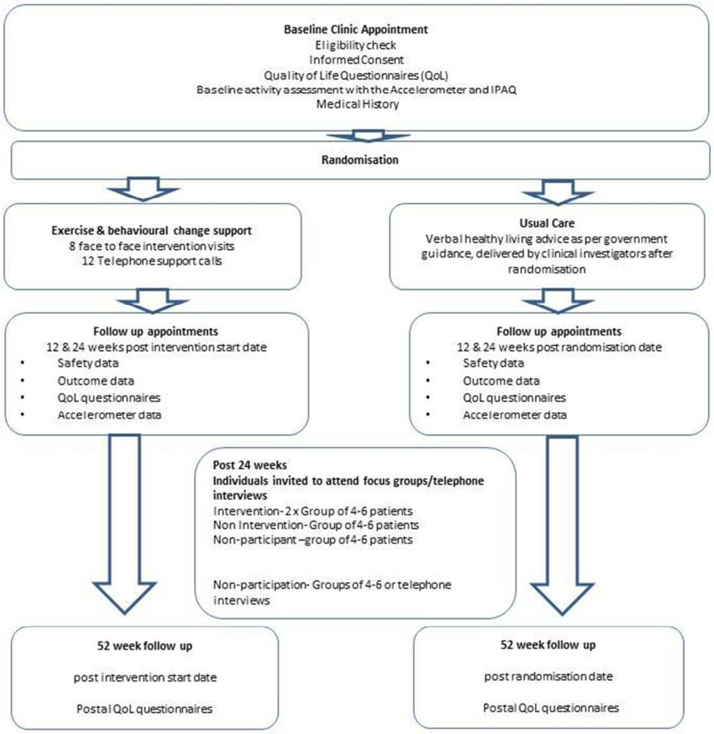

Consort diagram

254x190mm (96 x 96 DPI)

Treatment of fatigue with physical activity and behavioural change support in Vasculitis – study protocol for an open-label randomised controlled feasibility study.

Journal:	*BMJ Open*
Manuscript ID	bmjopen-2018-023769.R1
Article Type:	Protocol
Date Submitted by the Author:	19-Sep-2018
Complete List of Authors:	Harper, Lorraine; University of Birmingham<br>Morgan, Matthew; University of Birmingham<br>Chanouzas, Dimitrios; University of Birmingham<br>Caulfield, Hollie; University of Birmingham<br>Coughlan, Linda; University Hospitals Birmingham NHS Foundation Trust<br>Dean, Caroline; patient participant<br>Fletcher, Kate<br>Cramp, Fiona; The University of the West of England, Allied health professionals<br>Greenfield, Sheila; University of Birmingham, Primary Care Clinical Sciences<br>Hewitt, Catherine; University of Birmingham, Clinical Trials Unit<br>Ives, Natalie; University of Birmingham, BCTU<br>Jowett, Sue; University of Birmingham, Health Economics Unit<br>Daley, Amanda; Loughborough University, School of Sport, Exercise and Health Sciences
**Primary Subject Heading**:	Rheumatology
Secondary Subject Heading:	Renal medicine, Rehabilitation medicine
Keywords:	vasculitis, fatigue, exercise, anca, self-management

SCHOLARONE™
Manuscripts

For peer review only - http://bmjopen.bmj.com/site/about/guidelines.xhtml

Treatment of fatigue with physical activity and behavioural change support in Vasculitis – study protocol for an open-label randomised controlled feasibility study.

L. Harper[1], M.D. Morgan[1], D. Chanouzas[1], H Caulfield[2], L Coughlan[3], Caroline Dean[4], K Fletcher[2], F Cramp[5], S Greenfield[6], CA Hewitt[7], NJ Ives[7], S Jowett[6], A Daley[8].

1. Institute of Clinical Sciences, College of Medical and Dental Sciences, University of Birmingham, Birmingham B15 2TT, UK

2. Institute of Translational Medicine, College of Medical and Dental Sciences, University of Birmingham, Birmingham B15 2TT, UK

3. NIHR/Wellcome Trust Clinical Research Facility, UHB NHS Foundation Trust, Birmingham B15 2TH, UK

4. Patient Research Partner

5. Faculty of Health and Applied Sciences, University of the West of England Bristol, Bristol BS16 1DD, UK

6. Institute of Applied Health Research, College of Medical and Dental Sciences, University of Birmingham, Birmingham B15 2TT, UK

7. Birmingham Clinical Trials Unit, College of Medical and Dental Sciences, University of Birmingham, Birmingham B15 2TT, UK

8. School of Sport, Exercise and Health Sciences, Loughborough University, Loughborough LE11 3TU, UK

Address for correspondence

Professor Lorraine Harper, Institute of Clinical Sciences, College of Medical and Dental Sciences, University of Birmingham, Birmingham B15 2TT, UK

Email: L.harper@bham.ac.uk

For peer review only

Abstract

Introduction

Fatigue is a major cause of morbidity, limiting quality of life, in patients with ANCA-associated vasculitis (AAV). The aetiology of fatigue is multi-factorial; biological and psychosocial mediators, such as sleep deprivation, pain and anxiety and depression, are important, and may be improved by increasing physical activity. Current self-management advice is based on expert opinion and is poorly adhered to. This study aims to investigate the feasibility of increasing physical activity using a programme of direct contact and telephone support, to provide patient education, encourage behaviour self-monitoring and the development of an individual change plan with defined goals and feedback to treat fatigue compared to standard of care to inform the design of a large randomised controlled trial to test the efficacy and cost-effectiveness of this programme.

Methods and Analysis

Patients with AAV and significant levels of fatigue (patient self-report using multi-dimensional fatigue index score questionnaire ≥14) will be randomised in a 1:1 ratio to the physical activity programme supported by behavioural change techniques or standard of care. The intervention programme will consist of eight visits of supervised activity sessions and 12 telephone support calls over 12 weeks with the aim of increasing physical activity to the level advised by government guidelines. Assessment visits will be performed at baseline, 12, 24 and 52 weeks. The study will assess the feasibility of recruitment, retention, the acceptability, adherence and safety of the intervention and collect data on various assessment tools to inform the design of a large definitive trial. A nested qualitative study will explore patient experience of the trial through focus groups or interviews.

Ethics and Dissemination

All required ethical and regulatory approvals have been obtained. Findings will be disseminated through conference presentations, patient networks and academic publications.

Trial registration number: Prospectively registered ISRCTN 11929227 (registered 28.10.2016)

For peer review only

Strengths and limitations of this study

- Fatigue is a major cause of poor quality of life in patients with ANCA- associated vasculitis, and has no evidence base to direct treatment. This study will provide the evidence required to design a large randomised controlled trial to address whether physical activity improves fatigue in AAV patients.
- The study is limited by its size; as a feasibility study it has a small sample size and will not provide evidence of efficacy but will provide estimates to calculate sample size for a larger study

For peer review only

Introduction

Fatigue is a common symptom limiting quality of life in patients with a wide range of inflammatory and other chronic diseases including systemic vasculitis(1). Ninety two percent of patients with anti-neutrophil cytoplasm antibody associated vasculitis (AAV) consider fatigue as the most important symptom affecting their well-being(1, 2), and it has been causally linked to reduced social participation, social withdrawal and unemployment(3, 4). Despite the significance of the problem, there are currently no recommended therapies specifically for fatigue in patients with AAV. Current self-management advice provided by the National Institute for Health and Care Excellence (https://cks.nice.org.uk/tirednessfatigue-in-adults#!scenariorecommendation:1) and Arthritis Research UK (ARUK) (https://www.arthritisresearchuk.org/arthritis-information/daily-life/fatigue.aspx) is based on expert opinion and pragmatic advice without any underpinning evidence base. This is therefore, a major area of unmet need and has been identified as a priority research area by patients and ARUK.

The aetiology of fatigue is multi-factorial(5, 6) and related to a number of interacting central (such as reduced motivation and increased perception of effort) and peripheral (impaired muscle or cardiovascular function) biological and psychosocial mediators including sleep deprivation, pain, depression, lack of physical activity and reduced cardiovascular fitness, similar to other chronic diseases (7-9). (10). Only 47% of AAV patients participate in at least one hour of moderate or vigorous physical activity per week (unpublished audit of 100 patients) and patients are reluctant to increase physical activity often due to fatigue, or for fear of worsening their fatigue(11). Both central and peripheral components of fatigue need to be addressed in any successful treatment intervention.

Physical activity interventions improve sleep, quality of life, anxiety and depression in patients with chronic diseases, and have been shown to improve fatigue in the short term(12). However, studies with long term follow up often report no sustained increase in physical activity once support is discontinued(13). The addition of cognitive behavioural support (CBS), as commonly implemented in successful chronic disease self-management programmes(14), to a physical activity intervention may result in improved self-efficacy and a sustained increase in physical activity once the formal programme is completed(15-18).

For peer review only

Our programme, using direct contact and telephone support, will provide patient education, encourage behaviour self-monitoring and the development of an individual change plan with defined goals and feedback to increase physical activity. These interventions have not been investigated to treat fatigue in patients with systemic vasculitis. A pilot study of five AAV patients with fatigue suggested physical activity, supported by behavioural intervention, was an acceptable approach for patients with AAV and fatigue(19).

Aims and Objectives

The aim of this study is to assess the feasibility and acceptability of undertaking a phase III randomised controlled trial (RCT) of physical activity with behaviour change support including device assisted self-monitoring and telephone support with cognitive behavioural strategies compared to standard of care in patients with AAV.

The specific objectives of the study are to:

- Assess different recruitment methods to the trial e.g. where and how patients are identified and recruited
- Assess recruitment and retention rates in the trial
- Assess the acceptability of the intervention and the burden of trial participation on patients
- Assess adherence to the trial intervention
- Assess the safety of the intervention with respect to possible injury and deterioration in fatigue levels as a result of participating in a physical activity trial
- Assess the effect of trial participation on activity levels in patients in the standard care group
- Test procedures for the trial, including delivery of the standardised protocolled intervention and administration of trial questionnaires and other outcome measurement tools, and identify areas that may improve the design and implementation of the intervention
- Collect data on the variability of the outcome measures to inform a power calculation for a definitive trial
- Provide an estimate of the cost of implementing the intervention
- Collect feedback from patients on their experience of participating in the study

For peer review only

Methods

Overview of study design

This is a single centre, open-label randomised controlled feasibility study with a nested qualitative component. Feasibility will be determined by assessing recruitment, adherence to the intervention and retention rates. Acceptability and patient experience will be assessed through patient reported outcome questionnaires (PROQ), and an optional nested qualitative study consisting of either focus groups or semi-structured telephone interviews if insufficient numbers can be recruited to form focus groups..

Fifty participants will be randomised (via a computer generated programme) in a 1:1 ratio to the intervention or standard care for this population (Figure 1 study flow). The randomisation will be minimised by age (<65 vs. ≥65 years). Participants randomised to the intervention arm will complete eight supervised (one per week) and 12 telephone coaching sessions over 12 weeks. Outcomes will be assessed at baseline, 12, 24 and 52 weeks from the first exercise session for those in the intervention group and from randomisation for those in the standard care group.

Sample size

This is a feasibility study and therefore a formal sample size calculation has not been performed. The study is not designed or powered to detect a statistically significant difference in efficacy between the two treatment arms.

A pragmatic recruitment target of 50 participants has been chosen as we expect this number will be feasible within a single centre with a population of 250 patients and should be sufficient to provide estimates of the feasibility outcomes. A sample size of between 24 and 50 is recommended for pilot studies to estimate sample sizes for a full trial(20).

Patient Involvement

The study was designed in consultation with a range of patients. A patient research partner (CD) is part of the trial management group and helped design the study which was then discussed at patient support group meetings and with trustees of Vasculitis UK, the patient support charity. Feedback from these meetings was incorporated into the study design. Vasculitis UK has helped with patient recruitment and will help with dissemination of the study results.

Participants and recruitment

For peer review only

Patients will be recruited to the study via the dedicated vasculitis service at University Hospitals Birmingham NHS Foundation Trust (UHBNFT) or via response to study adverts placed in the Vasculitis UK newsletter or other hospital clinics. Confirmation of eligibility for the study will be with the patient's own clinician. Patients are eligible if they are 18 years of age or older, have a diagnosis of AAV as classified by the European Medicines Agency Algorithm (EMEA)(21), have been in remission for at least six months on the day of consent (defined by Birmingham Vasculitis Activity Score Version 3 (BVASv3)(22)=0 on day of consent AND prednisolone dose <7.5mg for 6 months) and have significant fatigue levels (multi-dimensional fatigue inventory (MFI)-20 general fatigue score $\geq$14). Exclusion criteria include inability to provide informed consent, inability or unwillingness to undertake physical activity, co-morbidities considered by their own clinician to contra-indicate an increase in physical activity and an inability to understand and complete questionnaires.

Standard Care Group

Patients randomised to the standard of care (control) group will receive their standard clinical care and will be provided with verbal activity advice as per UK government recommendations. Advice on activity will be given after randomisation and patients will be advised to visit the government physical activity guidelines website(23)

Intervention Group

The intervention is a physical activity and behavioural change support programme, plus standard care. The physical activity intervention will provide support for 12 weeks; weeks 1-8 will consist of weekly (where possible) direct contact in groups of 4-7 patients and additional individual telephone health coaching once weekly (where possible), then in weeks 9-12 telephone health coaching only will be provided. The intervention will be staged-adapted and participants will be encouraged to increase physical activity within their capabilities. Participants will be provided with personal activity self-monitoring devices, which are wrist worn accelerometers (Fitbit Model FB405BKL), providing individual feedback on daily physical activity. The supervised activity sessions will incorporate cognitive-behavioural strategies (e.g. goal setting, finding social support and understanding the costs/benefits of exercise etc.) to promote long-term participation in physical activity. The intervention includes education, monitoring and assessment of progress and teaches skills to improve self-efficacy.

Individual SMART (specific, measurable, achievable, realistic and timed) goals(24) will be set by the participants with guidance from the intervention facilitator and will focus on increasing activity

For peer review only

levels. The behavioural goal is for participants to achieve at least 30 minutes of moderate intensity activity, 5 days per week, ideally in bouts of 10 minutes, as per UK government recommendations, although any increase in physical activity or reduction in sedentary time will be viewed positively. The Fitbit activity self-monitor is supported with online web-interfaces that facilitate effective components of physical activity interventions (https://www.fitbit.com/login)(25).

A detailed facilitator intervention manual has been developed to support the supervised sessions and telephone coaching. In brief the physical activity intervention is designed to be pragmatic and accessible, taking into account exercise preferences and giving choices. During the direct contact sessions participants will be asked to complete short bouts (e.g. 5 × 3 minutes, with two-minute rest intervals) of low to moderate intensity aerobic exercise (e.g. stepping ergometer, cycle-ergometer, treadmill walking, rowing ergometer, arm-cranking) at 50%–69% of predicted maximum heart rate (220–age) or 12–14 on the Borg Ratings of Perceived Exertion Scale(26). Intensity will be monitored continuously during exercise training sessions. As the intervention progresses, participants will be encouraged to participate in longer periods of aerobic exercise (e.g. 5 × 4 minutes) or to take shorter rests between bouts.

Where appropriate, participants will also be introduced to exercises for strength and control, which will typically involve two to six different resistance exercises (e.g. wall press-ups, arm-curls, leg abduction, wall squats and/or regular squats, knee extensions, calf raises, sit-to-stand) each session. Body resistance, light weights and *Therabands* will be used to provide resistance and one to three sets of five to 20 repetitions will be performed, depending on level of disability and strength, as well as stage of the programme (exercises will be progressed according to individual capabilities and strength gains). Static stretching exercises for large skeletal muscle groups will be included in the sessions.

Participants will be encouraged to replicate the sessions at home supported by the telephone health coaching.

Using activity data collected from the Fitbit device from the preceding week, the intervention facilitator will assist participants to establish SMART goals(24). Data collected will be shared with the therapist, via either the internet dashboard or paper diary. Review of daily and weekly physical activity will be undertaken to identify bouts of moderate/vigorous physical activity and to negotiate change plans to increase bouts of moderate/vigorous physical activity.

For peer review only

During the telephone discussion, participants will be encouraged to use their activity profiles generated from their Fitbit device, with data collected and stored using the online dashboard or paper diaries, to promote self-determination and self-regulation to achieve personal goals and maintain activity. The therapist will review fatigue, the impact of physical activity on fatigue and the management of fatigue with the participant. The therapist will review activity plans and understanding, provide physical activity education, and help the patient generate activity maintenance plans and set new goals.

Study procedures

The study outcomes, measurement methods and assessments are summarised in table 1. Data is collected at baseline, and then at 12, 24 and 52 weeks from the first exercise session for those in the intervention group and from randomisation for those in the standard care group. Data on recruitment, number of activity sessions and telephone coaching sessions completed and drop outs will also be recorded.

For peer review only

Table 1 **Visit overview**

Visit	*Pre-baseline*	*Baseline*	*Baseline (+ 4 weeks)*	*Weeks 1-8*	*Weeks 9-12*	*Week 12**	*Week 24**	*Week 52**
Identification of eligible patients	*x*							
Eligibility check	*x*	*x*						
Valid informed consent		*x*						
Relevant medical history taken		*x*						
Randomisation (without patient present)			*x*					
*Activity advice telephone call (control arm only)***				*x*				
Exercise Visits (Intervention arm only)				*x*				
Weekly Telephone Coaching contacts (Intervention arm only)				*x*	*x*			
*Follow up clinic visits**$						*x*	*x*	
*Final assessment (postal questionnaires)**$								*x*
Quality of life questionnaires		*x*				*x*	*x*	*x*
Focus Groups or interviews (post intervention)					*x*	*x*	*x*	

**The timing of the follow up visits for intervention group are taken from intervention start date, not randomisation date; for the control group, the timing is taken from randomisation date.*

*** Control group have one telephone call in week 1 (post randomisation) to receive standard activity advice only.*

[$] 12 and 24 week follow up assessments can be completed up to -2 weeks before due date and up to 3 weeks after the due date

[$$] 52 weeks follow up assessment can be completed -2 weeks before due date and up to 8 weeks after the due date.

For peer review only

At the baseline, 12 and 24 week assessments blood pressure, height (baseline only), weight and waist circumference will be measured, disease type, medical and drug history (baseline only), use of immunosuppressive therapy and assessment of damage associated with AAV, using the vasculitis damage index(27) (baseline only) will be taken. Laboratory investigations, including full blood count and kidney function are measured at baseline. At each assessment visit, disease activity will be assessed using the BVASv3. Clinical outcomes and safety of the intervention, including muscle or bone injury, disease relapse and cardiovascular adverse clinical events will be collected at 12 and 24 weeks as self-reported by the participant. All serious adverse events will also be recorded. Participants will complete a suite of validated health related quality of life and fatigue questionnaires, which include Short Form-36 (SF-36)(28), ANCA Vasculitis questionnaire (AAV-PRO), EQ-5D(29), MFI-20(30), Pittsburgh Sleep Index(31), Bristol Rheumatoid Arthritis Fatigue- Multi-dimensional questionnaire (BRAF-MDQ)(32), Hospital Anxiety and Depression score (HADS)(33), Brief COPE questionnaire(34) and the International Physical Activity Questionnaire (IPAQ) long version at baseline and at 12, 24 and 52 weeks (at 52 weeks, the participants will be contacted by post and asked to complete the same questionnaires as completed at previous assessments and to return these by post or at next clinic visit). All participants will be asked to wear an accelerometer (GENEActive GATV01, Activinsights, Kimbolton UK) for 7 days at baseline and at 12 and 24 weeks to provide an objective measure of their activity.

In order to undertake an analysis of all the direct resources and costs required to deliver the intervention, a detailed collection of health economic data will be embedded within the trial.

Study outcomes and data analysis

The primary outcome of this feasibility trial is to determine if a full RCT comparing physical activity with behaviour change support versus standard care in patients with AAV is feasible. This decision will be made using a composite assessment of both quantitative and qualitative data based on a traffic light system using pre-defined stop-go criteria. The three criteria contributing to the traffic light system include (1) recruitment rates, defined as the proportion of eligible patients recruited into the study; (2) successful adherence to the intervention, defined as attendance at a minimum of 4 of the 8 visits for the face to face support, and acceptance of a minimum of 3 of the 4 telephone support calls in weeks 9-12 and (3) study drop-out, defined as complete withdrawal from the study, with no further data collected from the participant. The traffic light system comprises 1) **Green light**: recruitment rate >50%; adherence rate >75%, and drop-out rate <15% excluding those patients who drop-out or are unable to achieve adherence due to disease activity. If **all three** criteria are met, we

For peer review only - http://bmjopen.bmj.com/site/about/guidelines.xhtml

will proceed to a full trial with the protocol unchanged (unless there is a clear message from the qualitative work that would improve the protocol). 2) **Amber light:** recruitment rate 30-50%, adherence rate 50-75%, or drop-out rate 15-30% excluding those patients who drop-out or are unable to achieve adherence due to disease activity. If **one or more** of our amber light criteria are met, we will adapt the protocol in light of the result of the feedback from the qualitative work and our experience to improve whichever criteria are not at the “green-light” level before proceeding to full trial if possible. We will assess whether adaption of the protocol will require a further feasibility study or pilot study before progressing. 3) **Red light:** recruitment rate <30%, adherence rate <50%, or drop-out rate >30% excluding those patients who drop-out or are unable to achieve adherence for disease activity. If **one or more** of these criteria are met we would consider the current protocol not feasible and not progress to a full RCT.

The primary analysis to assess the stop-go criteria will be undertaken once all participants have completed the 24 week assessment and corresponding outcome data has been entered onto the study database and validated as being ready for analysis. Feasibility outcomes including the three components of the stop-go criteria will be analysed by pooling the two randomised groups and presenting overall estimates with 95% confidence intervals. Reasons for non-entry into the trial will be assessed, particularly in relation to the patient eligibility criteria and reasons for patient refusal. Data on patients who stop the intervention and/or who do not complete the trial (such as withdrawals and those lost to follow-up) will be collected throughout the trial to allow assessment of patient adherence and retention rates. Reasons for non-adherence and non-completion will be analysed descriptively.

All clinical and patient reported outcomes will primarily take the form of simple descriptive statistics (e.g. proportions and percentages, means and standard deviations) and where appropriate, point estimates of effects sizes (e.g. mean differences and relative risks) and associated 95% confidence intervals. Data return rates at each time point will be assessed along with data completeness of the various outcomes measures. This will inform the use of appropriate questionnaires for the primary outcome in a future study. The data will be analysed using the intention to treat principle, where patients are analysed in the treatment group to which they were randomised regardless of adherence to intervention or compliance with the protocol.

Detailed data collection of the resources required to deliver the intervention will allow a cost analysis to be undertaken to calculate the cost of implementing the intervention in a full RCT. The

For peer review only

mean cost of the intervention per patient will be estimated both as per usage in the study and also the full cost assuming full compliance. Data collected directly from the trial will determine the resources required for delivering the supervised exercise and telephone health coaching. Resources will include staff costs, any equipment/consumables needed, printed material, telephone call costs, staff training costs and infrastructure (e.g. room space). Information will be collected on number of patient contacts, length of time for face to face and telephone contacts and group size for the supervised exercise sessions. Standard unit costs for healthcare will be applied, with local costs sought from participating healthcare providers. Sensitivity analysis will estimate costs with changes to costing assumptions, e.g. staff grade, group size.

Nested qualitative study

Focus groups of 4-6 participants will be carried out to discuss their experience of the trial. Two groups will consist of people who participated in the trial and participated in the intervention, one group of people who participated and received standard of care; and the final group will be made up of people who did not wish to participate in the trial. Individuals who did not wish to participate in the trial will be contacted and invited to participate in a focus group to better understand the reasons for non-participation. If we cannot form one or more of the focus groups described, then patients will be invited to participate in 1:1 semi-structured telephone interviews The qualitative phase will be conducted by a researcher not involved with the rest of the study and will collect data on the experiences of the study and the intervention in more detail, and to gather suggestions for improvements to the design of a future RCT.

Focus groups and interviews will be recorded and transcribed verbatim. QSR NVivo 8 will be used for data management and data analysed using a framework approach(35), facilitated by the use of to identify freely emerging themes and categories. Those who did not participate in the trial will be invited to complete the same questionnaires as collected during the trial. These will be analysed as above using simple descriptive statistics.

Trial management and monitoring

The trial will be coordinated by the trial management group (TMG) (principal investigator, co-investigators, trial coordinator, physical activity trainer and statistical advisors) in conjunction with the NIHR/WT CRF. A Trial Steering Committee (TSC) consisting of the principal investigator as well as three independent consultants, a statistician and patient advocate not involved in the study will provide the overall supervision of the trial. The TSC will oversee trial progress, protocol compliance,

For peer review only

patient safety and review of updated information. As this is a feasibility study with short follow up, no Data Monitoring Committee will be formed, as agreed with the sponsor. The integrity of data entry will be ensured using a trial-specific Data Input Quality Control standard operating procedure (Trial Master File, University of Birmingham). The trial database will contain date of birth and sex. Data analyses will be undertaken on pseudo-anonymised datasets. All source data and original participant identities will be kept in a locked office in the trial site file only at UHB NHSFT or at BCTU.

Ethics

The protocol was reviewed and obtained a favourable opinion by the Health Research Authority (IRAS project ID 210364) on 29/09/16 and adopted on to the NIHR portfolio. All patients will be provided with verbal and written study information and written informed consent will be obtained before participation. The study will be undertaken at a single site, and local governance approval has been granted by UHB NHSFT. Steps have been taken to ensure patient welfare when designing this study. The study complies with the International Conference for Harmonisation of Good Clinical Practice guidelines and the Research Governance Framework for Health and Social Care. Any protocol amendments will be submitted to the sponsor and relevant regulatory bodies for approval prior to implementation and trial participants will be informed of any protocol modifications.

Sponsor

The study is sponsored by the University of Birmingham. The University of Birmingham holds public liability (negligent harm) and clinical trial (negligent harm) insurance policies, which apply to this trial. The sponsor has been involved in protocol design as well as the development of the case report form.

Funding

Funding for the study has been provided by Arthritis Research UK.

Dissemination policy

It is anticipated that the findings of this study will be published in peer-reviewed journals, presented at national conferences and that the results will be disseminated to all study participants who wish to be informed. It is anticipated that the results of this study will inform the design of a large RCT investigating the efficacy of increasing physical activity to treat fatigue in this population.

For peer review only

References

1. Grayson PC, Amudala NA, McAlear CA, Leduc RL, Shereff D, Richesson R, et al. Illness perceptions and fatigue in systemic vasculitis. Arthritis Care Res (Hoboken). 2013;65(11):1835-43.
2. Herlyn K, Hellmich B, Seo P, Merkel PA. Patient-reported outcome assessment in vasculitis may provide important data and a unique perspective. Arthritis Care Res (Hoboken). 2010;62(11):1639-45.
3. Basu N, McClean A, Harper L, Amft EN, Dhaun N, Luqmani RA, et al. Markers for work disability in anti-neutrophil cytoplasmic antibody-associated vasculitis. Rheumatology (Oxford). 2014;53(5):953-6.
4. Carpenter DM, Meador AE, Elstad EA, Hogan SL, DeVellis RF. The impact of vasculitis on patients' social participation and friendships. Clin Exp Rheumatol. 2012;30(1 Suppl 70):S15-21.
5. Basu N, McClean A, Harper L, Amft EN, Dhaun N, Luqmani RA, et al. Explaining fatigue in ANCA-associated vasculitis. Rheumatology (Oxford). 2013;52(9):1680-5.
6. McClean A, Morgan MD, Basu N, Bosch JA, Nightingale P, Jones D, et al. Physical Fatigue, Fitness, and Muscle Function in Patients With Antineutrophil Cytoplasmic Antibody-Associated Vasculitis. Arthritis Care Res (Hoboken). 2016;68:1332-9.
7. Jones DE, Gray JC, Newton J. Perceived fatigue is comparable between different disease groups. Qjm. 2009;102(9):617-24.
8. Hewlett S, Chalder T, Choy E, Cramp F, Davis B, Dures E, et al. Fatigue in rheumatoid arthritis: time for a conceptual model. Rheumatology (Oxford). 2011;50(6):1004-6.
9. Cleanthous S, Tyagi M, Isenberg DA, Newman SP. What do we know about self-reported fatigue in systemic lupus erythematosus? Lupus. 2012;21(5):465-76.
10. Newall C, Schinke S, Savage CO, Hill S, Harper L. Impairment of lung function, health status and functional capacity in patients with ANCA-associated vasculitis. Rheumatology. 2005;44:623-8.
11. Thorpe CT, DeVellis RF, Blalock SJ, Hogan SL, Lewis MA, DeVellis BM. Patient perceptions about illness self-management in ANCA-associated small vessel vasculitis. Rheumatology (Oxford). 2008;47(6):881-6.
12. Yang PY, Ho KH, Chen HC, Chien MY. Exercise training improves sleep quality in middle-aged and older adults with sleep problems: a systematic review. J Physiother. 2012;58(3):157-63.
13. Cramp F, Hewlett S, Almeida C, Kirwan JR, Choy EH, Chalder T, et al. Non-pharmacological interventions for fatigue in rheumatoid arthritis. Cochrane Database Syst Rev. 2013;8:CD008322.
14. Lorig KR, Holman H. Self-management education: history, definition, outcomes, and mechanisms. Ann Behav Med. 2003;26(1):1-7.
15. Hewlett S, Ambler N, Almeida C, Cliss A, Hammond A, Kitchen K, et al. Self-management of fatigue in rheumatoid arthritis: a randomised controlled trial of group cognitive-behavioural therapy. Ann Rheum Dis. 2011;70(6):1060-7.
16. Midtgaard J, Tveteras A, Rorth M, Stelter R, Adamsen L. The impact of supervised exercise intervention on short-term postprogram leisure time physical activity level in cancer patients undergoing chemotherapy: 1- and 3-month follow-up on the body & cancer project. Palliat Support Care. 2006;4(1):25-35.
17. Adamsen L, Quist M, Midtgaard J, Andersen C, Moller T, Knutsen L, et al. The effect of a multidimensional exercise intervention on physical capacity, well-being and quality of life in cancer patients undergoing chemotherapy. Support Care Cancer. 2006;14(2):116-27.
18. Tierney S, Mamas M, Woods S, Rutter MK, Gibson M, Neyses L, et al. What strategies are effective for exercise adherence in heart failure? A systematic review of controlled studies. Heart Fail Rev. 2012;17(1):107-15.

For peer review only

19. N Basu PP, S Lawton, M Greenwood, C Dent, K Kindness, E John, C Arthur, L West, MV Macfarlane, DM Reid, GT Jones, GJ Macfarlane. Multi-disciplinary Management of Fatigue in ANCA Associated Vasculitis: A Pilot Study. BSR. 2012.
20. Sim J, Lewis M. The size of a pilot study for a clinical trial should be calculated in relation to considerations of precision and efficiency. J Clin Epidemiol. 2012;65(3):301-8.
21. Watts R, Lane S, Hanslik T, Hauser T, Hellmich B, Koldingsnes W, et al. Development and validation of a consensus methodology for the classification of the ANCA-associated vasculitides and polyarteritis nodosa for epidemiological studies. Ann Rheum Dis. 2007;66(2):222-7.
22. Mukhtyar C, Lee R, Brown D, Carruthers D, Dasgupta B, Dubey S, et al. Modification and validation of the Birmingham Vasculitis Activity Score (version 3). Ann Rheum Dis. 2009;68(12):1827-32.
23. https://www.gov.uk/government/uploads/system/uploads/attachment_data/file/213741/dh_128146.pdf.
24. Doran G. There's a S.M.A.R.T. way to write management's goals and objectives. Manage Rev. 1981;70:35-6.
25. Hardcastle SJ, Taylor AH, Bailey MP, Harley RA, Hagger MS. Effectiveness of a motivational interviewing intervention on weight loss, physical activity and cardiovascular disease risk factors: a randomised controlled trial with a 12-month post-intervention follow-up. Int J Behav Nutr Phys Act. 2013;10:40.
26. Borg G. A category scale with ratio properties for intermodal and interindividual comparisons. In: Geissler H, Petzold P, eds. Psychophysical judgement and the process of perception; 1984:25-34.
27. Exley AR, Bacon PA, Luqmani RA, Kitas GD, Gordon C, Savage CO, et al. Development and initial validation of the Vasculitis Damage Index for the standardized clinical assessment of damage in the systemic vasculitides. Arthritis Rheum. 1997;40(2):371-80.
28. McHorney CA, Ware JE, Jr., Raczek AE. The MOS 36-Item Short-Form Health Survey (SF-36): II. Psychometric and clinical tests of validity in measuring physical and mental health constructs. Med Care. 1993;31(3):247-63.
29. Rabin R, de Charro F. EQ-5D: a measure of health status from the EuroQol Group. Ann Med. 2001;33(5):337-43.
30. Smets EM, Garssen B, Bonke B, De Haes JC. The Multidimensional Fatigue Inventory (MFI) psychometric qualities of an instrument to assess fatigue. J Psychosom Res. 1995;39(3):315-25.
31. Buysse DJ, Reynolds CF, 3rd, Monk TH, Berman SR, Kupfer DJ. The Pittsburgh Sleep Quality Index: a new instrument for psychiatric practice and research. Psychiatry Res. 1989;28(2):193-213.
32. Hewlett S, Dures E, Almeida C. Measures of fatigue: Bristol Rheumatoid Arthritis Fatigue Multi-Dimensional Questionnaire (BRAF MDQ), Bristol Rheumatoid Arthritis Fatigue Numerical Rating Scales (BRAF NRS) for severity, effect, and coping, Chalder Fatigue Questionnaire (CFQ), Checklist Individual Strength (CIS20R and CIS8R), Fatigue Severity Scale (FSS), Functional Assessment Chronic Illness Therapy (Fatigue) (FACIT-F), Multi-Dimensional Assessment of Fatigue (MAF), Multi-Dimensional Fatigue Inventory (MFI), Pediatric Quality Of Life (PedsQL) Multi-Dimensional Fatigue Scale, Profile of Fatigue (ProF), Short Form 36 Vitality Subscale (SF-36 VT), and Visual Analog Scales (VAS). Arthritis Care Res (Hoboken). 2011;63 Suppl 11:S263-86.
33. Zigmond AS, Snaith RP. The hospital anxiety and depression scale. Acta Psychiatr Scand. 1983;67(6):361-70.
34. Carver CS. You want to measure coping but your protocol's too long: consider the brief COPE. Int J Behav Med. 1997;4(1):92-100.

For peer review only

35. Gale NK, Heath G, Cameron E, Rashid S, Redwood S. Using the framework method for the analysis of qualitative data in multi-disciplinary health research. BMC Med Res Methodol. 2013;13:117.

Footnotes

Funding

This work was supported by Arthritis Research UK grant number 21199.

Ethical approval

The protocol was reviewed and obtained a favourable opinion by the Health Research Authority (IRAS project ID 210364) on 29/09/16 and adopted on to the NIHR portfolio

Competing interests

All authors have completed the ICMJE uniform disclosure form. No financial relationships with any organisations that might have an interest in the submitted work in the previous three years; no other relationships or activities that could appear to have influenced the submitted work have been declared by the authors.

Contribution

All authors (Morgan. Chanouzas, Caulfield, Coughlan, Dean, Fletcher, Cramp, Greenfield, Hewitt, Ives, Jowett, Daley, Harper) developed and reviewed the protocol. All authors (Morgan. Chanouzas, Caulfield, Coughlan, Dean, Fletcher, Cramp, Greenfield, Hewitt, Ives, Jowett, Daley, Harper) conceived the data analysis plan and the writing of this article. All authors (Morgan. Chanouzas, Caulfield, Coughlan, Dean, Fletcher, Cramp, Greenfield, Hewitt, Ives, Jowett, Daley, Harper) critically reviewed and edited drafts and approved the final version of the manuscript. They also had full access to all of the data (including statistical reports and tables) in the study and can take responsibility for the integrity of the data and the accuracy of the data analysis. The study guarantor is Harper.

Data sharing agreement – no additional data available

Provenance and peer review – not commissioned and externally peer reviewed

Acknowledgements

Trustees and patient members of Vasculitis UK are thanked for their helpful contributions which informed the development of the protocol. The work was carried out at the National Institute for Health Research (NIHR)/Wellcome Trust Birmingham Clinical Research Facility. The views expressed are those of the author(s) and not necessarily those of the NHS, the NIHR of the Department of Health.

Figure 1 Consort Diagram

For peer review only

For peer review only

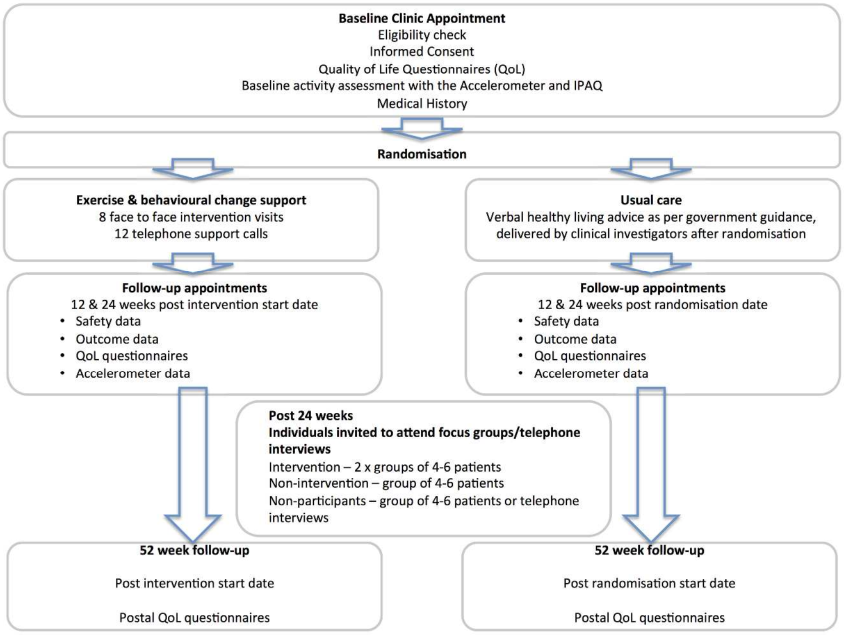

297x209mm (300 x 300 DPI)

For peer review only - http://bmjopen.bmj.com/site/about/guidelines.xhtml

SPIRIT

STANDARD PROTOCOL ITEMS: RECOMMENDATIONS FOR INTERVENTIONAL TRIALS

SPIRIT 2013 Checklist: Recommended items to address in a clinical trial protocol and related documents*

Section/item	Item No	Description	Addressed on page number
**Administrative information**			
Title	1	Descriptive title identifying the study design, population, interventions, and, if applicable, trial acronym	______1_______
Trial registration	2a	Trial identifier and registry name. If not yet registered, name of intended registry	______2______
	2b	All items from the World Health Organization Trial Registration Data Set	______2_______
Protocol version	3	Date and version identifier	Version 4.8 date 21/03/2018
Funding	4	Sources and types of financial, material, and other support	______14_______
Roles and responsibilities	5a	Names, affiliations, and roles of protocol contributors	______1______
	5b	Name and contact information for the trial sponsor	______14_______
	5c	Role of study sponsor and funders, if any, in study design; collection, management, analysis, and interpretation of data; writing of the report; and the decision to submit the report for publication, including whether they will have ultimate authority over any of these activities	______14_______
	5d	Composition, roles, and responsibilities of the coordinating centre, steering committee, endpoint adjudication committee, data management team, and other individuals or groups overseeing the trial, if applicable (see Item 21a for data monitoring committee)	_____13-14_____

For peer review only

For peer review only - http://bmjopen.bmj.com/site/about/guidelines.xhtml

**Introduction**			
Background and rationale	6a	Description of research question and justification for undertaking the trial, including summary of relevant studies (published and unpublished) examining benefits and harms for each intervention	_____4-5_______
	6b	Explanation for choice of comparators	_____6-7_______
Objectives	7	Specific objectives or hypotheses	______5_______
Trial design	8	Description of trial design including type of trial (eg, parallel group, crossover, factorial, single group), allocation ratio, and framework (eg, superiority, equivalence, noninferiority, exploratory)	______6_______
**Methods: Participants, interventions, and outcomes**			
Study setting	9	Description of study settings (eg, community clinic, academic hospital) and list of countries where data will be collected. Reference to where list of study sites can be obtained	______7_______
Eligibility criteria	10	Inclusion and exclusion criteria for participants. If applicable, eligibility criteria for study centres and individuals who will perform the interventions (eg, surgeons, psychotherapists)	______7_______
Interventions	11a	Interventions for each group with sufficient detail to allow replication, including how and when they will be administered	______7-9_____
	11b	Criteria for discontinuing or modifying allocated interventions for a given trial participant (eg, drug dose change in response to harms, participant request, or improving/worsening disease)	_____11, 13-14__
	11c	Strategies to improve adherence to intervention protocols, and any procedures for monitoring adherence (eg, drug tablet return, laboratory tests)	______7-9_____
	11d	Relevant concomitant care and interventions that are permitted or prohibited during the trial	________7______
Outcomes	12	Primary, secondary, and other outcomes, including the specific measurement variable (eg, systolic blood pressure), analysis metric (eg, change from baseline, final value, time to event), method of aggregation (eg, median, proportion), and time point for each outcome. Explanation of the clinical relevance of chosen efficacy and harm outcomes is strongly recommended	_____11-13____
Participant timeline	13	Time schedule of enrolment, interventions (including any run-ins and washouts), assessments, and visits for participants. A schematic diagram is highly recommended (see Figure)	________10______

For peer review only - http://bmjopen.bmj.com/site/about/guidelines.xhtml

Sample size	14	Estimated number of participants needed to achieve study objectives and how it was determined, including clinical and statistical assumptions supporting any sample size calculations	_______6______
Recruitment	15	Strategies for achieving adequate participant enrolment to reach target sample size	_______6______

Methods: Assignment of interventions (for controlled trials)

Allocation:

Sequence generation	16a	Method of generating the allocation sequence (eg, computer-generated random numbers), and list of any factors for stratification. To reduce predictability of a random sequence, details of any planned restriction (eg, blocking) should be provided in a separate document that is unavailable to those who enrol participants or assign interventions	_______6______
Allocation concealment mechanism	16b	Mechanism of implementing the allocation sequence (eg, central telephone; sequentially numbered, opaque, sealed envelopes), describing any steps to conceal the sequence until interventions are assigned	_______6______
Implementation	16c	Who will generate the allocation sequence, who will enrol participants, and who will assign participants to interventions	_______6______
Blinding (masking)	17a	Who will be blinded after assignment to interventions (eg, trial participants, care providers, outcome assessors, data analysts), and how	_____N/A______
	17b	If blinded, circumstances under which unblinding is permissible, and procedure for revealing a participant's allocated intervention during the trial	_____N/A______

Methods: Data collection, management, and analysis

Data collection methods	18a	Plans for assessment and collection of outcome, baseline, and other trial data, including any related processes to promote data quality (eg, duplicate measurements, training of assessors) and a description of study instruments (eg, questionnaires, laboratory tests) along with their reliability and validity, if known. Reference to where data collection forms can be found, if not in the protocol	______9-11____
	18b	Plans to promote participant retention and complete follow-up, including list of any outcome data to be collected for participants who discontinue or deviate from intervention protocols	______9-11____

For peer review only - http://bmjopen.bmj.com/site/about/guidelines.xhtml

Data management	19	Plans for data entry, coding, security, and storage, including any related processes to promote data quality (eg, double data entry; range checks for data values). Reference to where details of data management procedures can be found, if not in the protocol	_____13-14_
Statistical methods	20a	Statistical methods for analysing primary and secondary outcomes. Reference to where other details of the statistical analysis plan can be found, if not in the protocol	_____11-13_____
	20b	Methods for any additional analyses (eg, subgroup and adjusted analyses)	______13_______
	20c	Definition of analysis population relating to protocol non-adherence (eg, as randomised analysis), and any statistical methods to handle missing data (eg, multiple imputation)	______11-12____
**Methods: Monitoring**			
Data monitoring	21a	Composition of data monitoring committee (DMC); summary of its role and reporting structure; statement of whether it is independent from the sponsor and competing interests; and reference to where further details about its charter can be found, if not in the protocol. Alternatively, an explanation of why a DMC is not needed	______14_______
	21b	Description of any interim analyses and stopping guidelines, including who will have access to these interim results and make the final decision to terminate the trial	______N/A______
Harms	22	Plans for collecting, assessing, reporting, and managing solicited and spontaneously reported adverse events and other unintended effects of trial interventions or trial conduct	________11______
Auditing	23	Frequency and procedures for auditing trial conduct, if any, and whether the process will be independent from investigators and the sponsor	______13-14____
**Ethics and dissemination**			
Research ethics approval	24	Plans for seeking research ethics committee/institutional review board (REC/IRB) approval	________14______
Protocol amendments	25	Plans for communicating important protocol modifications (eg, changes to eligibility criteria, outcomes, analyses) to relevant parties (eg, investigators, REC/IRBs, trial participants, trial registries, journals, regulators)	________14______

For peer review only - http://bmjopen.bmj.com/site/about/guidelines.xhtml

Consent or assent	26a	Who will obtain informed consent or assent from potential trial participants or authorised surrogates, and how (see Item 32)	________7_______
	26b	Additional consent provisions for collection and use of participant data and biological specimens in ancillary studies, if applicable	_______N/A_______
Confidentiality	27	How personal information about potential and enrolled participants will be collected, shared, and maintained in order to protect confidentiality before, during, and after the trial	_______14________
Declaration of interests	28	Financial and other competing interests for principal investigators for the overall trial and each study site	_______17________
Access to data	29	Statement of who will have access to the final trial dataset, and disclosure of contractual agreements that limit such access for investigators	_______17________
Ancillary and post-trial care	30	Provisions, if any, for ancillary and post-trial care, and for compensation to those who suffer harm from trial participation	_______14______
Dissemination policy	31a	Plans for investigators and sponsor to communicate trial results to participants, healthcare professionals, the public, and other relevant groups (eg, via publication, reporting in results databases, or other data sharing arrangements), including any publication restrictions	_______14________
	31b	Authorship eligibility guidelines and any intended use of professional writers	_______N/A_______
	31c	Plans, if any, for granting public access to the full protocol, participant-level dataset, and statistical code	_______N/A_______
**Appendices**			
Informed consent materials	32	Model consent form and other related documentation given to participants and authorised surrogates	supplementary data __________
Biological specimens	33	Plans for collection, laboratory evaluation, and storage of biological specimens for genetic or molecular analysis in the current trial and for future use in ancillary studies, if applicable	_______N/A_______

*It is strongly recommended that this checklist be read in conjunction with the SPIRIT 2013 Explanation & Elaboration for important clarification on the items. Amendments to the protocol should be tracked and dated. The SPIRIT checklist is copyrighted by the SPIRIT Group under the Creative Commons "Attribution-NonCommercial-NoDerivs 3.0 Unported" license.

For peer review only - http://bmjopen.bmj.com/site/about/guidelines.xhtml
